# WHO Scientific Working Group on monitoring and management of bacterial resistance to antimicrobial agents.

**DOI:** 10.3201/eid0101.950111

**Published:** 1995

**Authors:** F C Tenover, J M Hughes


### WHO Scientific Working Group on Monitoring and Management of Bacterial Resistance to Antimicrobial Agents

Antibacterial resistance is a global clinical and public health problem that has emerged with alarming rapidity in recent years and undoubtedly will increase in the near future. Resistant bacteria do not respect national borders, and developments in the remote locations can have an impact throughout the world. Resistance is a problem in the community as well as in health care settings, where transmission of bacteria is greatly amplified, in both developed and developing countries. Because multiple drug resistance is a growing problem, physicians are now confronted with infections for which there is no effective therapy. The morbidity, mortality, and financial costs of such infections pose an increasing burden for health care systems worldwide, but especially in countries with limited resources.

The Division of Communicable Diseases at the World Health Organization, Geneva, Switzerland, recently convened a Scientific Working Group to address the problem of drug-resistant bacterial infections. From November 29 to December 2, 1994, participants from 23 countries reviewed and discussed scientific data on the nature and costs of drug resistance; recent national and global trends; approaches to limiting the emergence and spread of resistance in community and institutional settings; and strategies to strengthen local, national, and global surveillance. Participants included representatives from clinical medicine, public health, the clinical laboratory, and the biomedical research arenas and from the pharmaceutical industry.

The Working Group formulated a series of recommendations to address these issues at local, national, and international levels. The recommendations placed emphasis on enhanced surveillance of drug resistance through usage of WHONET software, increased monitoring and improved usage of antimicrobial drugs in human, veterinary, and animal husbandry settings, improved laboratory diagnostic capacity, standardization and quality control of laboratory methodology, professional and public education, development of new drugs and assessment of alternative therapeutic modalities, assessment of vaccine development and delivery priorities related to antimicrobial resistance, better implementation of infection control measures, and evaluation of prevention strategies.

The Working Group plans to release its final report in the spring.

**Fred C. Tenover**
National Center for Infectious Diseases
Centers for Disease Control and Prevention
Atlanta, Georgia, USA

**James M. Hughes**
National Center for Infectious Diseases
Centers for Disease Control and Prevention
Atlanta, Georgia, USA

